# A Study on Micropipetting Detection Technology of Automatic Enzyme Immunoassay Analyzer

**DOI:** 10.1038/s41598-018-24145-0

**Published:** 2018-04-10

**Authors:** Zhiwu Shang, Xiangping Zhou, Cheng Li, Sang-Bing Tsai

**Affiliations:** 1 Tianjin Key Laboratory of Modern Mechatronics Equipment Technology, Tianjin Polytechnic University, Tianjin, 300387 China; 2 Zhongshan Institute, University of Electronic Science and Technology of China, Guangdong, 528400 China; 3 Economics and Management College, Civil Aviation University of China, Tianjin, 300300 China

## Abstract

In order to improve the accuracy and reliability of micropipetting, a method of micro-pipette detection and calibration combining the dynamic pressure monitoring in pipetting process and quantitative identification of pipette volume in image processing was proposed. Firstly, the normalized pressure model for the pipetting process was established with the kinematic model of the pipetting operation, and the pressure model is corrected by the experimental method. Through the pipetting process pressure and pressure of the first derivative of real-time monitoring, the use of segmentation of the double threshold method as pipetting fault evaluation criteria, and the pressure sensor data are processed by Kalman filtering, the accuracy of fault diagnosis is improved. When there is a fault, the pipette tip image is collected through the camera, extract the boundary of the liquid region by the background contrast method, and obtain the liquid volume in the tip according to the geometric characteristics of the pipette tip. The pipette deviation feedback to the automatic pipetting module and deviation correction is carried out. The titration test results show that the combination of the segmented pipetting kinematic model of the double threshold method of pressure monitoring, can effectively real-time judgment and classification of the pipette fault. The method of closed-loop adjustment of pipetting volume can effectively improve the accuracy and reliability of the pipetting system.

## Introduction

With the improvement of living standards, people pay more and more attention to health and food safety. ELISA analyzer as *in vitro* diagnostic analysis of clinical laboratory instruments necessary, is widely used in clinical detection, epidemiology, immunology, endocrinology, food safety and other fields^1,2^, has become an important metheds of detection and prevention of disease. Immunoassay technology has also received great attention: Dianping Tan *et al*. reported a near-infrared light-to-UV light-mediated photoelectrochemical (PEC) aptasensing platform and PCE immunoassay technique based on quantum dots^3–5^. It can accurately detect the target antigen when the sample concentration is as low as pg mL-1, and truly realizes “a drop of blood to see all diseases”; Lin’s group designed a novel simultaneously visual and photoelectrochemical (PEC) immunosensing system for rapid sensitive detection of Aflatoxin B1(AFB1) in foodstuff^6,7^, it can rapid detection of small molecules at low sample concentrations. Micropipette detection technology is one of the key technologies for automating immune analysis. It involves a number of core technologies such as liquid level detection, pipetting fault monitoring, and pipetting result detection, and it is of great importance in ensuring the reliability of immune detection, improving detection efficiency, and reducing reagent usage.

In the past few decades, scholars at home and abroad have put forward many methods to determine the process of absorbing liquids. Michael Kaplit^8^ analyzed the linear regression analysis of a pressure curve before the end of the suction process. According to the standard deviation and the critical pressure value to determine the shortage problem of sample size, reduced the amount of data acquisition, Masaaki Takeda^9^ and other people have classified and analyzed the problem of the needle and designed the special pressure discrimination circuit. The sampling values of different sampling periods are fed into different circuits, and compared with the threshold values set by them, the accurate discrimination of the plugging needle is realized. The advantage is that the dynamic response is fast, but the circuit structure is more complicated. The advantage is that the dynamic response is fast, but the circuit structure is more complicated. The above two methods are only to judge a certain problem, and the evaluation of the liquid transfer process is not comprehensive. The normal pressure curve error band was obtained by J. L. Camenisch^10^ through the experiment, and the measured pressure curve in the absorption liquid was compared with the normal adsorption curve, and the total evaluation of the liquid removal process was realized. However, when the absorbability and the sample concentration are not at the same time, the acquisition of the error band requires a large number of repetitive experiments, which are less versatile. A qualitative evaluation model of the liquid removal process was established by means of grey clustering analysis by Zhu Lian-Qing and other people^11,12^. It can accurately the fault identification and classification, but the complex evaluation model, and the failure phenomenon appears no effective solutions.

In order to meet the reliability and efficiency of the micro-displacement fluid, The fault of pipetting is monitored in real time by the pressure monitoring, and after the pipetting fault occurs, image recognition is used to quantitatively identify the actual pipetting amount in the pipetting tip, and the pipetting deviation value is fed back to the automatic pipetting motion control system. The automatic pipetting control system compensates and adjusts the pipetting volume. The method that combined the real-time of pressure monitoring with quantitative identification by image processing, fault monitoring and closed-loop adjustment of the pipetting system are realized to reduce the pipetting error and increase the reliability and precision of the pipetting system, thereby ensuring the reliability of the immunoassay.

## System Design

**Figure 1:**
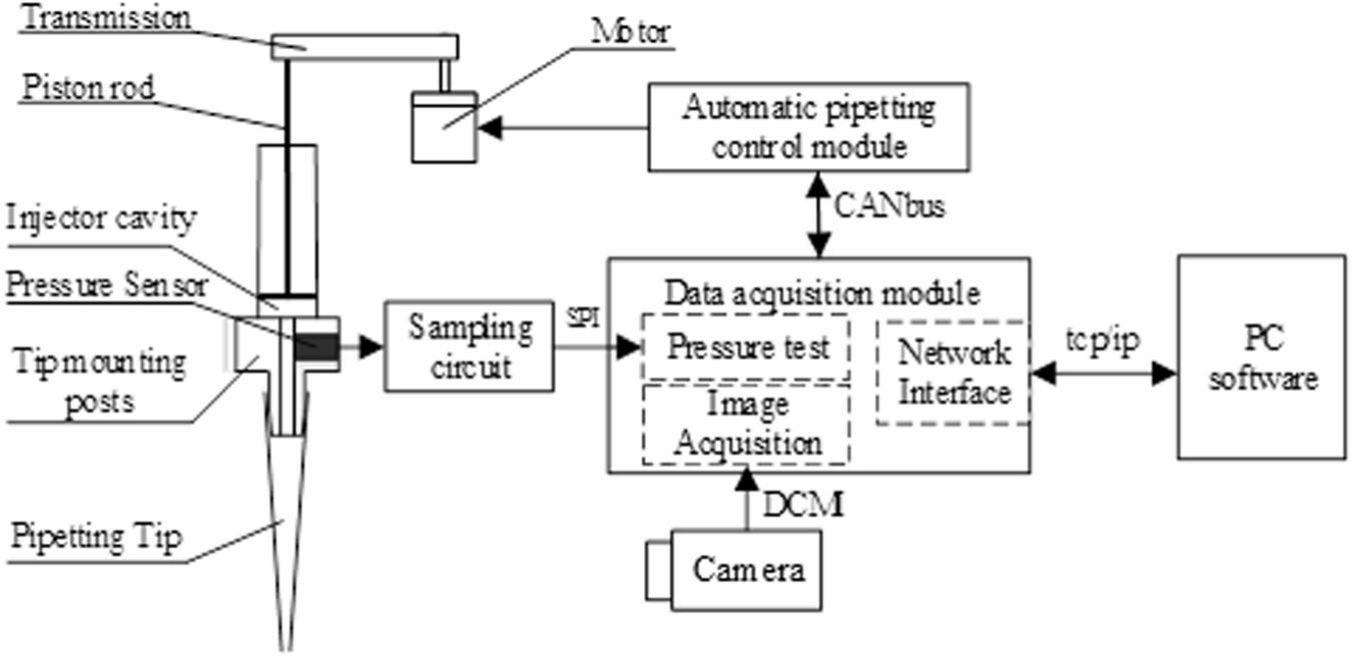
Block diagram of trace liquid detection system.

The whole microflow system can be divided into three modules: automatic liquid transfer control module, data detection and acquisition module, and upper computer moving liquid image processing module. The data detection and acquisition module also serves as the information exchange hub for the whole system, and CAN communicate with the automatic liquid transfer control module to establish Ethernet communication with the upper computer. The block diagram of the trace shift detection system is shown in Fig. 1.

The data acquisition module is divided into two sub-modules: pressure detection module and image acquisition module. Using STM32F767IGT6 based on CORTEX-M7 kernel, the core processor of data acquisition module is adopted. The processor has the resources of digital camera interface DCMII, image accelerator, Ethernet MAC interface, double precision FPU floating-point arithmetic, DMA processor, etc., which satisfies the requirements of the system. The whole system running in the uc/OS-III real-time operating system, for the process provides a reliable and efficient operation environment.

Pressure detection module: the pressure monitoring module is responsible for testing the liquid level of reagent and the pressure monitoring of the suction process. As shown in Fig. 1, the pressure detection module includes pressure sensor, sampling circuit and pressure signal acquisition and processing MCU. The pressure sensor is MS5611-01BA03, installed on the side wall of the suction mounting column, to detect the pressure in the fluid chamber. The sensor is integrated with 24 A/D conversion modules and outputs the data in I2C/SPI interface with A conversion frequency of up to 20 Mhz.

Image acquisition module: the image acquisition module is processed after receiving the command, and processed by Ethernet transmission. The whole module includes image acquisition and image transmission, in which the image acquisition terminal adopts CMOS camera OV5640. The camera has a 500 W hd pixel and can still reach 20 frames per second when setting the maximum pixel 1600 × 1400 output. The image is output with DCMII interface, matching with the digital camera interface of STM32F7 processor, which can reach speeds of up to 54 M/s.

Image recognition module of liquid displacement: the upper computer software USES OpenCV for image processing and the calculation of liquid displacement. OpenCV inherits a large number of digital image processing function packages, which can greatly shorten the development cycle^13^.

**Figure 2:**
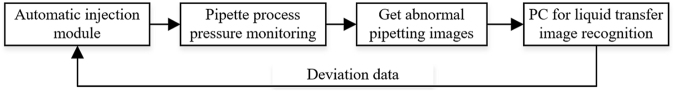
The closed-loop control flow chart of the microfluid-displacement detection system.

The flow chart of the whole test system is shown in Fig. 2. Liquid level testing is carried out before the liquid is removed. The pressure sensor is used to detect the internal pressure of the liquid. After the anomaly, obtain the image after the removal of the liquid. Sent via Ethernet is the highest level computer software for image processing, fluid volume calculation, and will feedback the pipetting abnormal values to the automatic sampling module, which can adjust the error, the implementation of trace closed-loop adjustment.

## Dynamic Monitoring of the Pressure Process

During the measurement of the change of the internal pressure of the fluid in the fluid during the removal process, the abnormal conditions such as low suction, leakage, air suction and blockage occurred in the process of the real-time detection of liquid removal. If these exceptions are captured in time, the corresponding processing algorithm can be corrected. Therefore, the monitoring of air pressure in piston can greatly reduce the uncertainty in the automatic liquid treatment, making the system more reliable.

### Establishment of pressure model

In the process of removing fluid, the fluid is inhaled and discharged in order to continuously balance the differential pressure inside and outside the piston. Under static equilibrium conditions, the pressure in the piston, the suction fluid and the atmospheric pressure meet the following relationship:

1 $${P}_{I}+{P}_{L}={P}_{I}+(\rho \times g\times h)={P}_{0}$$

Where *P*_*I*_ is the gas pressure of the piston, P0 is the atmospheric pressure, *P*_*L*_ is in the suction pressure of the liquid gravity, and *P*_*L*_* = ρ × g × h*, *ρ* is liquid density, *g* is gravitational constant, *h* is the height of the suction liquid. The change of pressure in the chamber of the pipettes is:

2 $${P}_{I}={P}_{0}-\rho \times g\times h$$

Equation 2 shows that if atmospheric pressure *P*_0_ is stable, the pressure *P*_*I*_ in the piston can directly reflect the changes in the liquid inside the suction head.

The characteristics of liquid inhalation and the amount of liquid absorbed can be indirectly reflected by the change of air pressure. But as a result of the sensor precision in the process of actual sample, sample liquid density and the complexity of the fault phenomena, this method cannot to quantitative detection of sample quantity, only through change the cavity pressure in the body of the sample, the qualitative judgment of whether the sample qualified, and through the anomaly characteristics of the fault types are classified.

The pressure in the pipette chamber during the pipetting process is in a dynamic equilibrium process and is affected by the ambient temperature, the viscosity and density of the liquid, and the speed of the piston rod. The immunoassay is generally completed in a constant temperature environment. Due to the diversity of sample reagents, the viscosity and density are uncontrollable factors, but the speed is a direct factor that causes pressure changes. To simplify the pressure detection model and improve the monitoring efficiency, this scheme normalizes the influence factor of uncontrollable factors on pressure to *β*, and establishes the pressure model of the pipetting process by the pipetting speed. The 7-stage S-type acceleration/deceleration control algorithm used in the automatic pipetting system has a motion model such as formula (3):

3 $$v(t)=\{\begin{array}{c}{v}_{0}+\frac{1}{2}h{t}^{2};t\in [0,{t}_{1})\\ {v}_{1}+{a}_{m}(t-{t}_{1});t\in [{t}_{1},{t}_{2}),{v}_{1}={v}_{0}+\frac{1}{2}h{{t}_{1}}^{2}\\ {v}_{2}+{a}_{m}(t-{t}_{2})-\frac{1}{2}h{(t-{t}_{2})}^{2};t\in [{t}_{2},{t}_{3}),{v}_{2}={v}_{1}+{a}_{m}({t}_{2}-{t}_{1})\\ {v}_{3};t\in [{t}_{3},{t}_{4}),{v}_{3}={v}_{2}+{a}_{m}({t}_{3}-{t}_{2})-\frac{1}{2}h{({t}_{3}-{t}_{2})}^{2}\\ {v}_{4}-\frac{1}{2}h{(t-{t}_{4})}^{2};t\in [{t}_{4},{t}_{5}),{v}_{4}={v}_{3}\\ {v}_{5}-{a}_{m}(t-{t}_{4});t\in [{t}_{5},{t}_{6}),{v}_{5}={v}_{4}-\frac{1}{2}h{({t}_{5}-{t}_{4})}^{2}\\ {v}_{6}-{a}_{m}(t-{t}_{5})+\frac{1}{2}h{(t-{t}_{5})}^{2};t\in [{t}_{6},{t}_{7}),{v}_{6}={v}_{5}-{a}_{m}({t}_{6}-{t}_{5})\end{array}$$

Where *v*_0_ is the starting speed, *h* is acceleration, and *a*_*m*_ is the acceleration. The velocity motion model is transformed into a sample volume change model. It can be exppressed as:

4 $${\rm{\Delta }}V=\frac{s}{l}\times \frac{1}{i}\times {\int }_{0}^{t}v(t)dt$$

In the formula, *s* is the capacity of the sampler, *l* is the sampler range, *i* is the transmission ratio of transmission mechanism. According to the ideal gas law, the pressure inside the pipette chamber can be calculated as:

5 $$P\times V=n\times R\times T$$

In the formula, *n* is the quantity of matter; *T* is the absolute temperature; *R* is the molar gas constant. The pressure change in the cavity is:

6 $${\rm{\Delta }}P=n\times R\times T/({V}_{0}+{\rm{\Delta }}V)-{P}_{0}$$

Collaborate on the formula to establish a normalized pressure model:

7 $${P}_{I}={P}_{0}-{\int }_{0}^{t}({\rm{\Delta }}P-\beta \times \rho \times g\times h)dt$$

**Figure 3:**
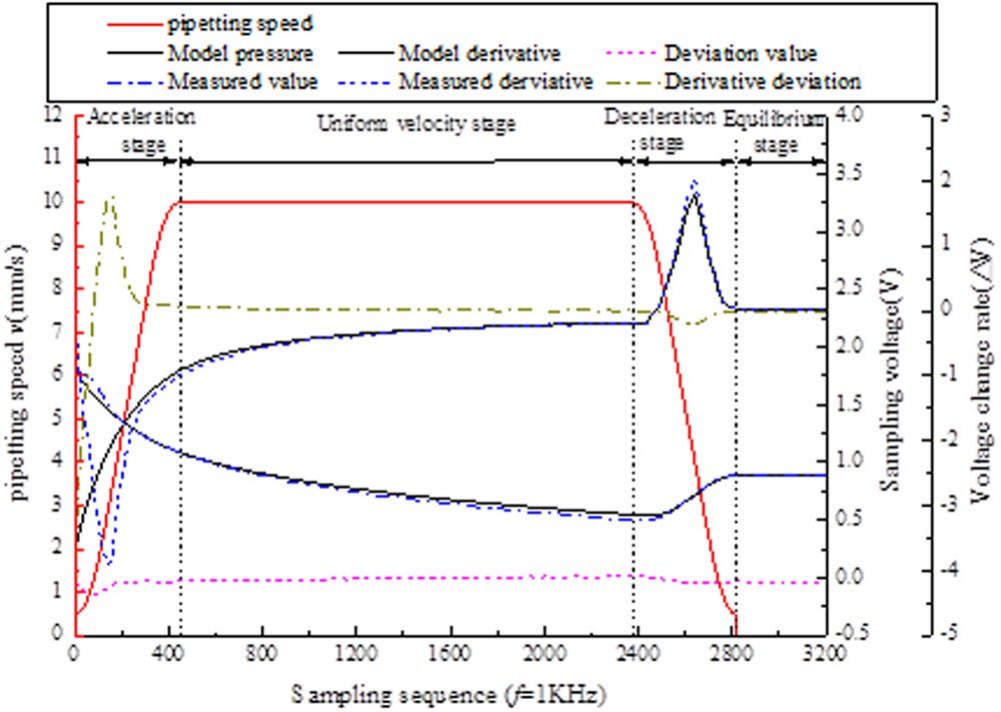
Comparison of the theoretical model with the actual sampling pressure.

The pressure data of the normal pipetting experiment were collected, and the normalized influence factor β was calibrated by bias correction to improve the accuracy of the pressure model. The detection pressure and calibration model characteristic curve during the normal process of pipetting is shown in Fig. 3.

1. Acceleration phase: The pressure gas in the chamber is stretched and the pressure is reduced obviously. However, due to the infiltration phenomenon of the liquid, there is a great difference between the pressure model and the actual pipetting pressure.
2. The uniform speed phase: The pressure in the cavity changes smoothly, and the pressure model can better reflect the actual pressure change.
3. In the deceleration phase: the injection speed slows down, but the pressure difference between the inside and outside of the chamber is still very large, and the liquid suction speed gradually slows down and finally maintains the balance. The pressure model at this stage can better reflect the actual pressure changes.
4. In the balancing phase: the pipetting operation is completed and the pressure enters the static equilibrium phase. The pressure model at this stage can better reflect the actual pressure value.

From the comparison of the theoretical pressure curve and the monitoring value of the actual aspiration process pressure, it can be seen that although there is liquid infiltration in the acceleration phase, there is a certain difference between the model and the actual. However, as a whole, the normalized pressure model better reflects the pressure changes in the pipetting process.

### Kalman dynamic filtering

Because of the electromagnetic interference and mechanical vibration of the circuit, the output signal of the pressure sensor contains a lot of noise. In order to improve the accuracy of the sample results, the results are guaranteed and the sampling results need to be filtered. Because the output of pressure sensor in the system needs to be calculated and determined in real time, the filtering algorithm adopted by the system must have high real-time performance. And the amount of information of the microfluid-displacement system is time varying, the influence quantity is uncertain, and the output quantity is determined. Kalman filtering algorithm is widely used in solving the problem of small number of cases, randomness, etc.^14,15^. This scheme adopts the dynamic filtering algorithm based on the first order kalman filter to process the sampling results of the pressure sensor to reduce the noise interference in the sensor output.

The kalman filter consists of two main processes: estimation and correction. Forecast process mainly use time update equation based on the current state of a prior estimate and timely forward calculated value of the current state variables and the error covariance estimation, state structure for the next time a priori estimate; The calibration process is responsible for feedback, and the improved posterior estimation of the current state is established based on the prior estimate of the prediction process and the current measurement variables. Such a process, we call it: the prediction - calibration process. The corresponding estimation algorithm is called: prediction - correction algorithm^16^. The time update equation and status update equation of discrete kalman filter are given.

8 $${\hat{x}}_{k}=A{\hat{x}}_{k-1}+B{\hat{U}}_{k-1}$$

9 $${P}_{k}^{-}=A{P}_{k-1}\,{A}^{T}+Q$$

Status update equation:

10 $${K}_{k}={P}_{k}^{-}{H}^{T}{(H{P}_{k}^{-}{H}^{T}+R)}^{-1}$$

11 $${\hat{x}}_{k}={\hat{x}}_{k}^{-}+{K}_{k}({z}_{k}-H{\hat{x}}_{k}^{-})$$

12 $${P}_{k}=(I-{K}_{k}H){P}_{k}^{-}$$

The various functions are:

Equation (8): extrapolate state variables forward;

Equation (9): forward calculation error covariance;

Equation (10): calculate the kalman gain;

Formula (11): updated by observation variable *z*_*k*_;

Formula (12): update error covariance.

**Figure 4:**
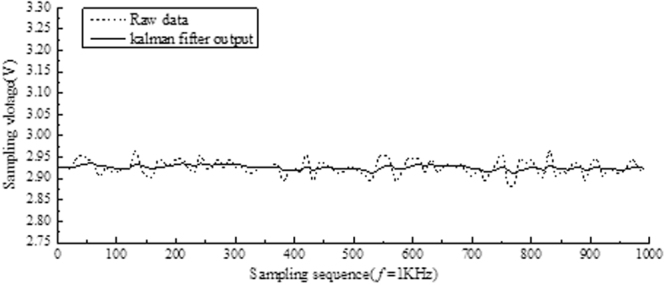
Filtering results and the original data comparison chart.

The collected pressure data is fed into a first order kalman filter, and the original data is obtained with the kalman filter. As shown in Fig. 4. The test results show that kalman filter effectively reduces interference.

### Dynamic pressure monitoring of double threshold method

There is a certain difference in the concentration of different samples during the removal process, and there is a small fluctuation in the movement speed of the mechanism during the removal process. These factors will affect the final pressure change. In order to improve the application range of the whole system, the first derivative double factor of the pressure value and pressure curve is used as the anomaly criterion.

The following conclusions can be obtained by analyzing the pressure curve:

1. In the process of absorption liquid pressure, the first derivative curve of two obvious peaks, respectively in the sample in the early stages of acceleration and deceleration.
2. In the stage of constant absorption, the pressure changes gently, but the overall pressure difference is large, and the first derivative of the pressure changes little.
3. At the end of the sample, the cavity pressure less than atmospheric pressure inside body, in theory can be gained through balancing formula (1), if there are less suck air leakage, etc., the value will be varied with the target, can determine whether pipetting qualified.

To this end, the sample process is divided into four stages to establish the corresponding criteria for judging characteristics:

1. The acceleration/deceleration sample stage (First order derivative extremum method): as a standard sample first derivative peak value of the pressure in the model as the evaluation standard, peak threshold range of sample set to monitor the pressure in the process of change.
2. Uniform sample stage (First derivative threshold method): thresholds are set using the first derivative of the pressure in the pipetting model to monitor changes in pressure during the pipetting process.
3. The sample balance phase (Pressure deviation threshold method): through the cavity pressure difference value *in vivo* and *in vitro* anti push fluid absorption quantity, calculating the with the target sample quantity deviation, deviation threshold setting, determine the amount of liquid is qualified.

**Figure 5:**
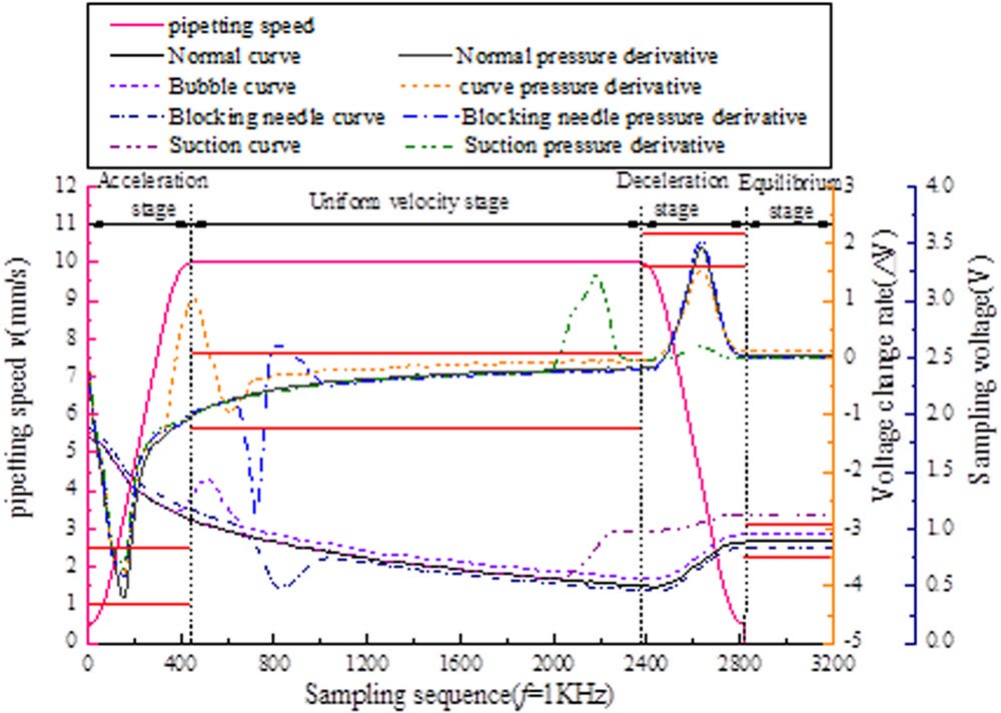
Comparison of the characteristics of abnormal phenomena in the migration fluid.

The pressure of the pipetting process is monitored by the parameters of each stage, and the pipetting process is considered qualified only when the parameters of each stage of the injection process are within the threshold range. Set up the common phenomenon of abnormal phenomenon of pipetting and classify the faults by means of polyclassification. In the pressure sensor sampling frequency *f* = 1KHz, the pipette common faults are tested experimentally, and the segmented dual-threshold detection results are shown in Fig. 5.

The monitoring results shows that the segmented dual-threshold diagnostic method can effectively monitor and classify the abnormal phenomenon of pipetting, and the pipette process pressure model established based on the pipetting operation kinematics model can achieve good results only by performing a few experiments and parameter calibration.

## Quantitative Detection of Fluid Displacement Based on Image Processing

The pressure monitoring method can effectively determine the fault phenomenon in the process of removing liquid. However, due to a large number of factors affecting the displacement anomaly, the pressure method can only determine the qualitative determination of the liquid transfer process. In order to solve the deficiency of the reliability of trace liquid and pressure monitoring method, this scheme proposes a complementary solution: the quantitative identification method of image method. The method of image processing is used to test the liquid capacity of the suction head after the sample and get the specific suction value. The automatic sample system is given specific guidance to eliminate the abnormality after the occurrence of the migration anomaly^17^. The image recognition processing algorithm consists of the following three parts:

1. Extraction of liquid reagent characteristics;
2. Identification of pixel boundary geometry parameters of liquid reagent;
3. Calculation of scale conversion and displacement volume.

### Background comparison method to extract the characteristics of the reagent

**Figure 6:**
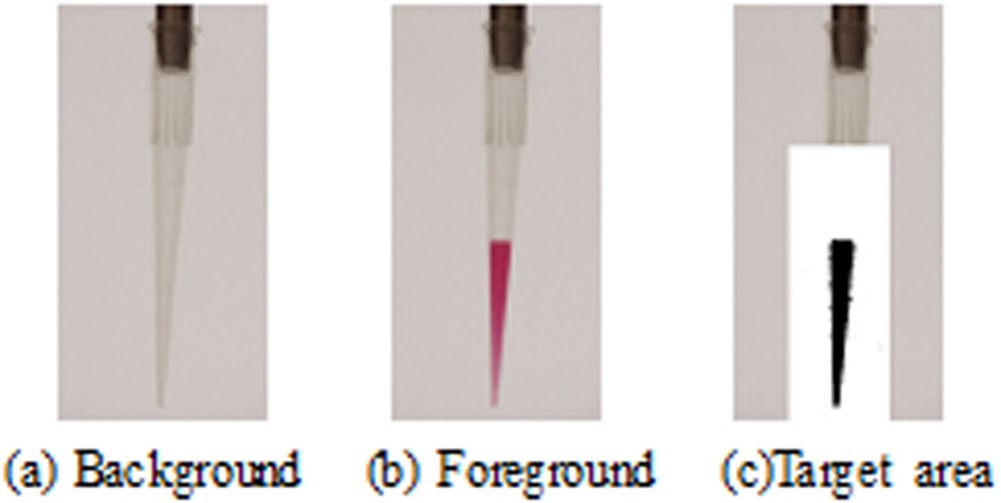
Liquid feature extraction effect map.

Background comparison method refers to the background of the image obtained without the suction fluid, and compares the image with the background image to the region of the reagent in the suction head after removing the fluid. The lighting environment in the removal system is relatively stable, and the position of the suction head for each reagent can be predicted, so that only the specific target area needs to be processed. In order to improve the reliability of background information, the median filter method is used to obtain the sample reagent regional background. The image of five unaspirated liquid is collected continuously, and the image is obtained by taking the mean of each pixel of the image to the extreme value. After the removal of the fluid, the image of the pipe-sucking area was obtained, and the difference region was extracted by threshold method. Meanwhile, in order to reduce the computation and improve the processing efficiency, only the image processing of the area of the piped suction head is carried out. The processing results are shown in Fig. 6.

After extracting the actual characteristics of the pipetting, the planar area of the pipetting tip after the injection was acquired. However, due to the mapping effect of the liquid color on the pipette tip wall after the pipette tip is added, more interference noises appear on the edge of the pipetting reagent. According to the characteristics of the noise distribution is relatively small, scattered, the image is open operation (the first corrosion after the expansion), so that the edge noise has been effectively filtered.

### Migration volume identification algorithm

**Figure 7:**
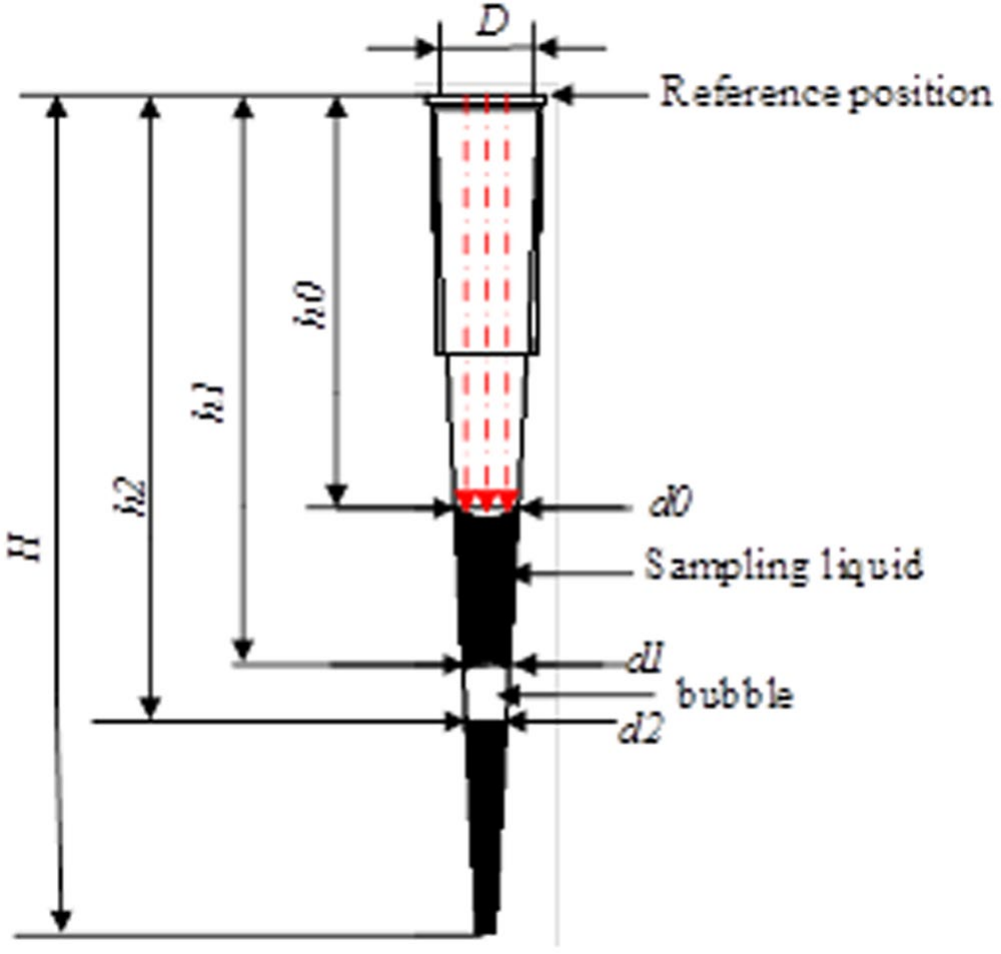
Calculation model of pipetting volume.

After extracting the liquid in the suction head, the liquid pixel area of the suction head is obtained, which is equivalent to the semi-section of the liquid in the suction head. With the known suction head mounting end plane as the reference position, the liquid level of the liquid at the suction head is highly h0. The volume of liquid in the suction head can be obtained according to the geometrical shape parameters of the suction head. There may be bubbles in the liquid, and the volume model of the liquid volume is shown in Fig. 7.

**Figure 8:**
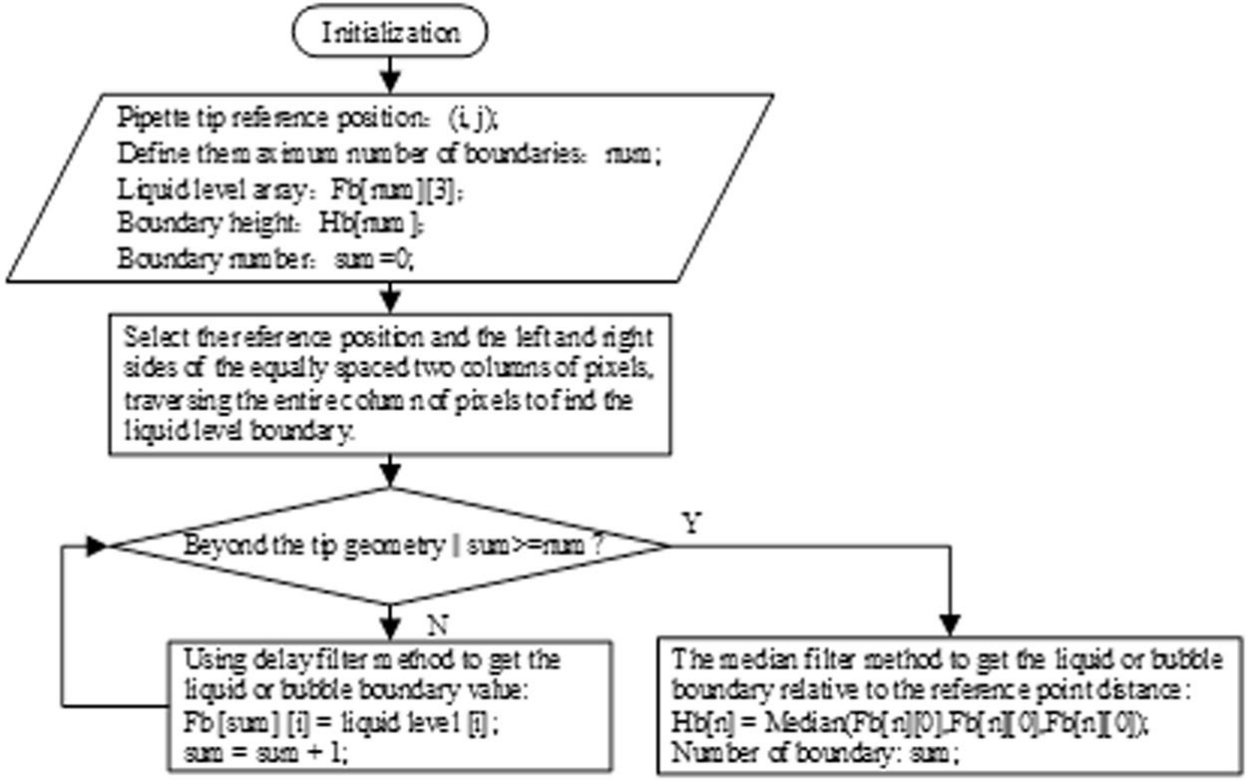
Algorithm of liquid surface boundary acquisition.

The three liquid face values were filtered by the median filter method. In order to avoid interference at the same time, when the liquid surface boundary, using delay filter: when the continuous acquisition three pixel values for the liquid, with the first liquid collection value as the par value, or will not be included in the results. The flowchart of its algorithm is shown in Fig. 8. Through the above algorithm, the liquid level *h*_0_ will be obtained, and if there are bubbles in the liquid, then the bubble boundary will be obtained (*h*_*n*_*, h*_*n* + *1*_) and *n* (1, 2, 3, …).

### Scale conversion and liquid displacement calculation

After the above processing, the geometrical parameters of the moving liquid reagent in the image are obtained. But what you get here is the pixel unit, which doesn’t reflect the actual size. Scale changes are needed to get the actual size of a single pixel. This scheme with known the size of a standard block, placed in the same plane with suction, obtaining images at this time, to identify the standard block width *w* in image pixel number *n*, then the pixel size factor *e* is:

13 $$e=w/n$$

According to move the geometry characteristic parameters of liquid suction, suction head volume of liquid in myopia into cone volume V_1_, the bubble volume as the volume of frustum of a cone. V_n_ (n: the number of air bubbles), the final volume of liquid in the suction V = V_1_ − ∑V_n_. The formula of liquid calculation is obtained by integrating the parameters of the incoming sample and the scaling factor.

14 $$\{\begin{array}{c}V=\frac{1}{12}\pi ({{d}_{0}}^{2}(H-{h}_{0})\\ -\sum _{i=1}^{n}({h}_{2i}-{h}_{2i-1})({{d}_{2i-1}}^{2}+{{d}_{2i}}^{2}+{d}_{2i-1}\,\ast \,{d}_{2i}))\,\ast \,{e}^{3}\\ {d}_{i}=\frac{(H-{h}_{i})\,\ast \,D}{H};i\in (0,1,2,\mathrm{...})\end{array}$$

Where: *H* is the overall height of the suction head; D is the suction head.

## Experimental Verification and Error Compensation

### Experimental verification of image method

According to the experimental requirements of biochemical analysis, the volumetric determination experiment of 100 ul volume was used as the maximum absorbing capacity. The calibration reference to the international standard ISO 8655-6^18^, in which the specified point of inspection is 10 ul, 50 ul and 100 ul, with the capacity to allow error and repetition rate as the standard for inspection. Using weighing method to check the additive results. The test configuration is as follows:

1. Experimental platform: a fully automatic enzyme-linked immunoassay analyzer was developed independently, with high accuracy^19,20^, and the test system was loaded on this instrument.
2. Measuring medium: animal serum.
3. Measuring equipment: 250 ul suction head and 0.01 mg electronic balance for quality determination.
4. The calculation formula of repeatability error of the measurement results is formulas (15) and (16) respectively.

15 $$CV=(s/{x}_{l})\,\ast \,100 \% $$

16 $$error=\frac{\mathop{x}\limits^{\_}-{x}_{l}}{{x}_{l}}\,\ast \,100 \% $$

In the formula: *x*_*l*_ is the target sample quantity, *S* is the standard deviation: $s=\sqrt{\sum _{i=1}^{n}{({x}_{i}-\overline{x})}^{2}/(n-1)}$.

Measurement method 1: by weighing method, the sample size was 10 ul, 50 ul and 100 ul respectively. Each range was measured 8 times, and the results of each measurement were calculated. The test is used to detect the stability of the sample system. The experimental data is shown in Table 1.

**Table 1:** Fixed value stability test results table.

Actual value/mg	1	2	3	4	5	6	7	8	Average value/mg	CV/%	error/%
9.98	10.99	11.18	11.05	11.21	11.18	11.15	11.11	11.13	11.12	1.12	9.94
50.2	54.44	54.81	54.57	54.97	54.82	54.75	54.67	55.03	54.76	0.74	8.77
99.82	108.8	109.14	108.65	109.46	109.17	109.03	108.86	109.57	109.09	0.37	8.43

**Figure 9:**
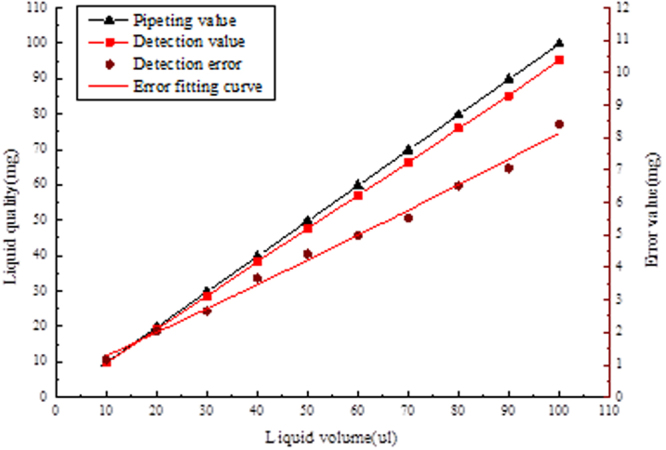
Data graph of slinear stability test.

Measurement method 2: select 10 measuring points including the point of inspection to measure, and each test point will be tested 10 times, then take the mean. The experimental data is shown in Fig. 9. The error source of the model is analyzed by this experiment.

The analysis results show that the approximate method is adopted in the image recognition algorithm model, and the liquid surface of the actual sample reagent in the suction head is not the complete plane. Due to the gravity effect of certain sag, the approximate algorithm simplifies the model, but brings some errors. Reduce the measuring error approach are: 1. The image processing algorithm for further processing, the sampling points by liquid level position curve fitting out sag, again into the three-dimensional volume, subtract this part in the moving fluid volume calculation formula sag volume; 2. The deviation between measured value and actual displacement value is analyzed by experimental method. The error compensation curve is obtained by fitting the function to compensate the measured value.

### Error compensation and comprehensive test

By improving the image algorithm to make the depression curve fitting, the obtained image has a reflection phenomenon, which can’t obtain the better fitting effect. At the same time, the image algorithm becomes more complex and will affect the efficiency of the whole detection. Therefore, the scheme adopts the method of linear compensation for error to reduce the detection error. The least squares linear fitting was performed using the detection of liquid displacement deviation in the experimental data. Get the error fitting equation:

17 $${V}_{actual}=-0.0763{V}_{{detection}}+0.309$$

The error fitting function is compensated to the calculation function of the detection value, and the calculation formula of the displacement after compensation is obtained:

18 $$\{\begin{array}{c}V=(1-0\mathrm{.0763})\,\ast \,(\frac{1}{12}\pi ({{d}_{0}}^{2}(H-{h}_{0})\\ -\sum _{i=1}^{n}({h}_{2i}-{h}_{2i-1})({{d}_{2i-1}}^{2}+{{d}_{2i}}^{2}+{d}_{2i-1}\,\ast \,{d}_{2i}))\,\ast \,{e}^{3})+0.309\\ {d}_{i}=\frac{(H-{h}_{i})\,\ast \,D}{H};i\in (0,1,2,\mathrm{...})\end{array}$$

**Figure 10:**
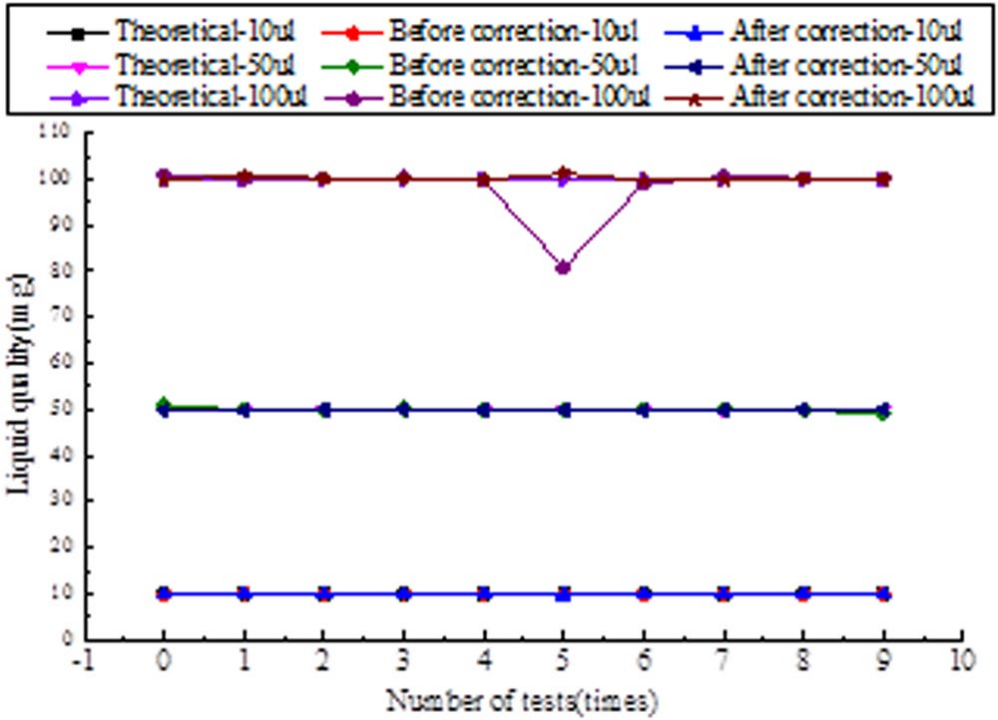
Data comparison chart of before and corrected.

**Figure 11:**
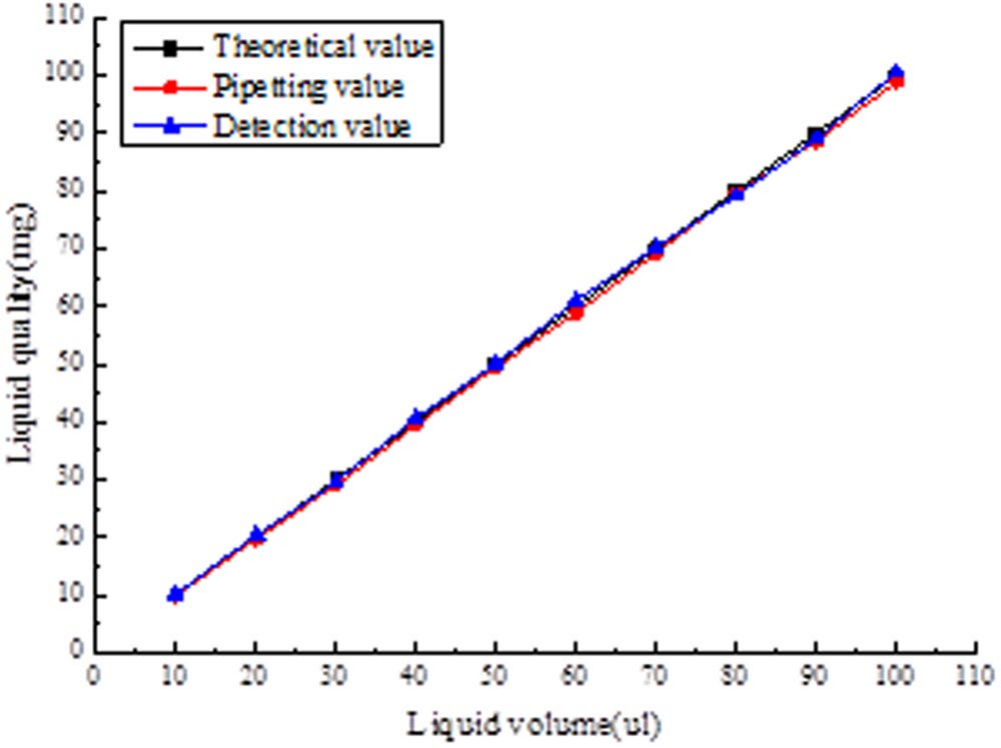
Test data after compensation.

After the error compensation, the micro-injection system was tested in accordance with test mode 1 and test mode 2 again. In the test, the automatic sample system and the liquid displacement detection system are tested in the test, when the pressure method determines the abnormal time of the liquid, the image detection system can be activated. The detection module of image method will be used to transmit the deviation value to the sample system. Automatic sampling is used to calibrate the suction fluid until the test is qualified and record the pre-calibration and post-calibration data. The test data is shown in Fig. 10. The experimental results of test mode 2 after error compensation are shown in Fig. 11 respectively.

The accuracy and repeatability errors of the detection system of 10 ul, 50 ul, 100 ul and the image test system can be calculated by formula (14) (15), The experimental results shown in Table 2.

**Table 2:** System accuracy and repeatability error.

Test point	10 ul	50 ul	100 ul
Pipette system accuracy error (%)	±1.8(5.6)	±1.28(3.2)	±1.15(2.8)
Pipette system reproducibility error (%)	±1.31(2.48)	± 0.56(1.25)	± 0.40(1.02)
Detect system accuracy error (%)	± 1.63(9.94)	± 0.99(8.77)	± 0.85(8.43)
Detect system reproducibility error (%)	± 0.82(1.12)	± 0.48(0.74)	± 0.32(0.37)

(Hint: The data statistics format is: after calibration (before calibration)).

The experimental results show that the compensation function greatly reduces the measurement error of the image test module, and the detection module can effectively detect the automatic sampling error, and guide the automatic sample module to reduce the sampling error in a certain range, so as to make the amount of liquid transfer reach the standard.

## Summary

1. The pressure-learning model of the kinematics model based on the kinematics model can better reflect the change of pressure in the lumen of the pipe-fluid during the removal process. Good monitoring results can be obtained by a small amount of experimental calibration. The method can be used to determine and classify the fault of the moving fluid effectively.
2. Background correlation method combined with moving liquid suction geometric characteristics of the fluid volume image recognition algorithm can realize the quantitative measurement of moving fluid volume and compensate the error of linear fitting in the detection accuracy was further improved.
3. Liquid removal process monitoring and liquid removal result quantitative detection of the process evaluation system can effectively monitor the migration process. In this paper, the automatic sampling module is used to correct the deviation of the moving fluid. The accuracy and reliability of the micro-displacement system are improved without affecting the operation efficiency of the trace liquid, and the dosage of reagent is saved.
